# Supplementary figures and images for: Cytosolic EpCAM cooperates with H-Ras to regulate epithelial to mesenchymal transition through ZEB1
Source: PLoS One. 2023 May 16;18(5):e0285707. doi: 10.1371/journal.pone.0285707 (PMC10187930; doi:10.1371/journal.pone.0285707)

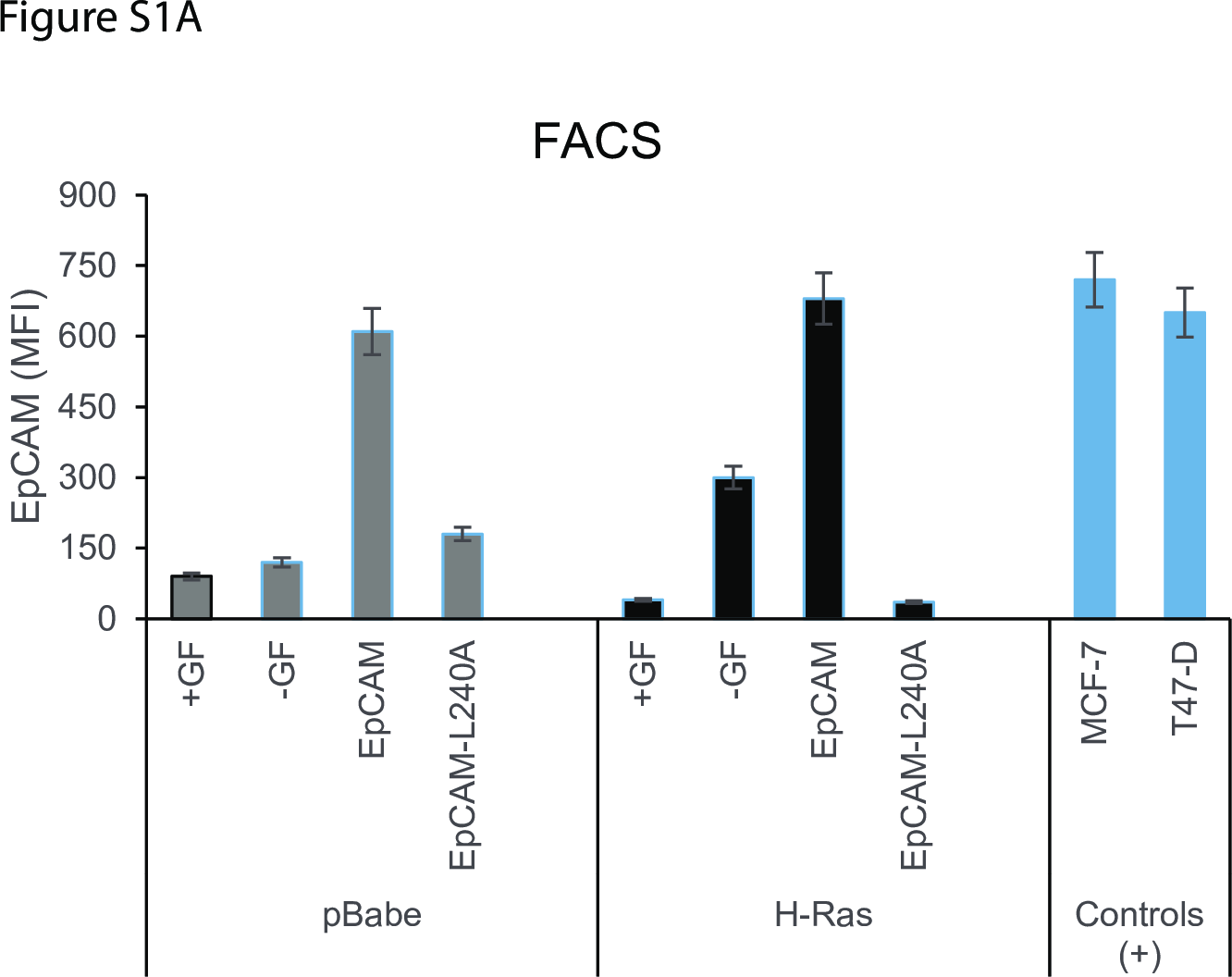

Supplement: S1 Fig — A. Flow cytometry analysis of surfaced stained EpCAM. MCF10-A cells transduced with Ras and EpCAM were analyzed using flow cytometry to distinguish between WT and cytosolic EpCAM. MCF-7 and T47-D cells were used as controls to monitor comparable expression of exogenous EpCAM. Cytosolic EpCAM is not detected in surface staining of cells. B. EpCAM closely correlated genes are expressed together in Ras positive epithelial cells. NCI-60 cancer cell lines (GSE5846) with Ras mutations were analyzed for expression of EpCAM and its “Nearest Neighbors” using GENE-E software. (ZIP) [file pone.0285707.s001.zip › Figure S1A.tif]

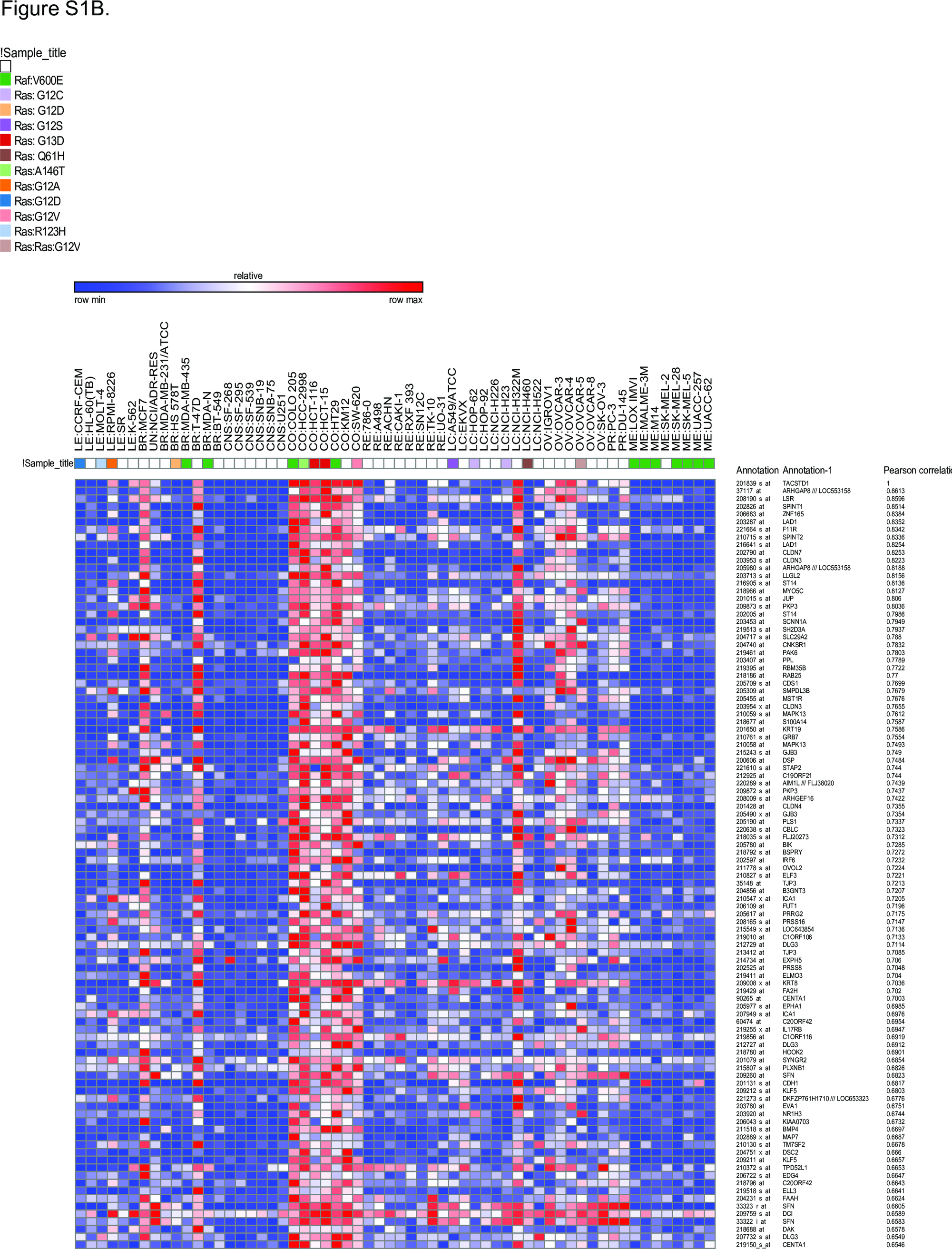

Supplement: S1 Fig — A. Flow cytometry analysis of surfaced stained EpCAM. MCF10-A cells transduced with Ras and EpCAM were analyzed using flow cytometry to distinguish between WT and cytosolic EpCAM. MCF-7 and T47-D cells were used as controls to monitor comparable expression of exogenous EpCAM. Cytosolic EpCAM is not detected in surface staining of cells. B. EpCAM closely correlated genes are expressed together in Ras positive epithelial cells. NCI-60 cancer cell lines (GSE5846) with Ras mutations were analyzed for expression of EpCAM and its “Nearest Neighbors” using GENE-E software. (ZIP) [file pone.0285707.s001.zip › Figure S1B.tif]

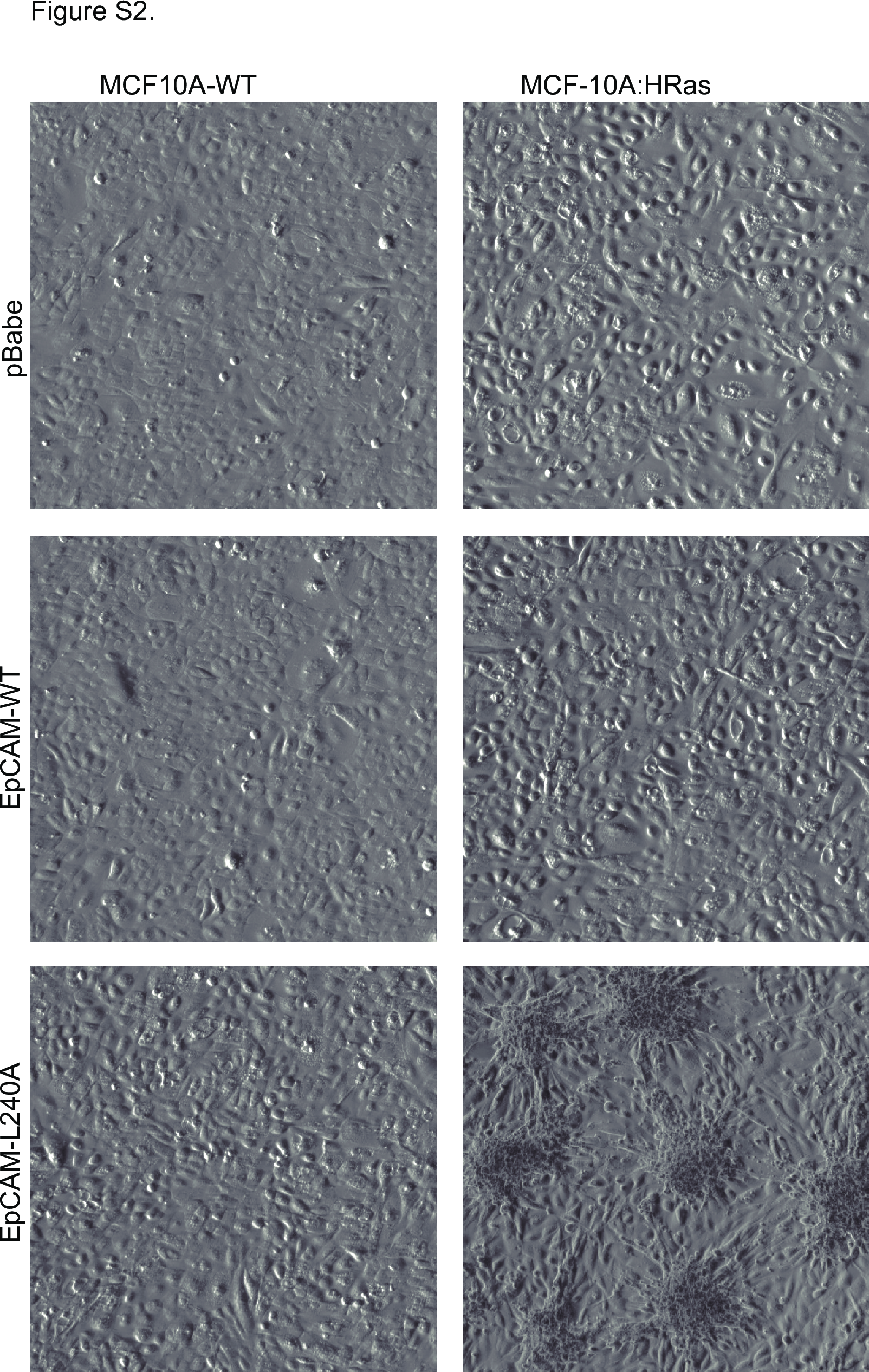

Supplement: S2 Fig — MCF10A cells with vector, Ras, and EpCAM. Flat monolayer growth can be seen in MCF10-pBabe and EpCAM WT-cells. Protruding bi-layer like growth (see Discussion) can be seen in MCF10A-L240A and MCF10A-Ras-pBabe and EpCAM-WT cells. Totally altered morphology was observed in Ras-EpCAM-L240A cells. (TIF) [file pone.0285707.s002.tif]

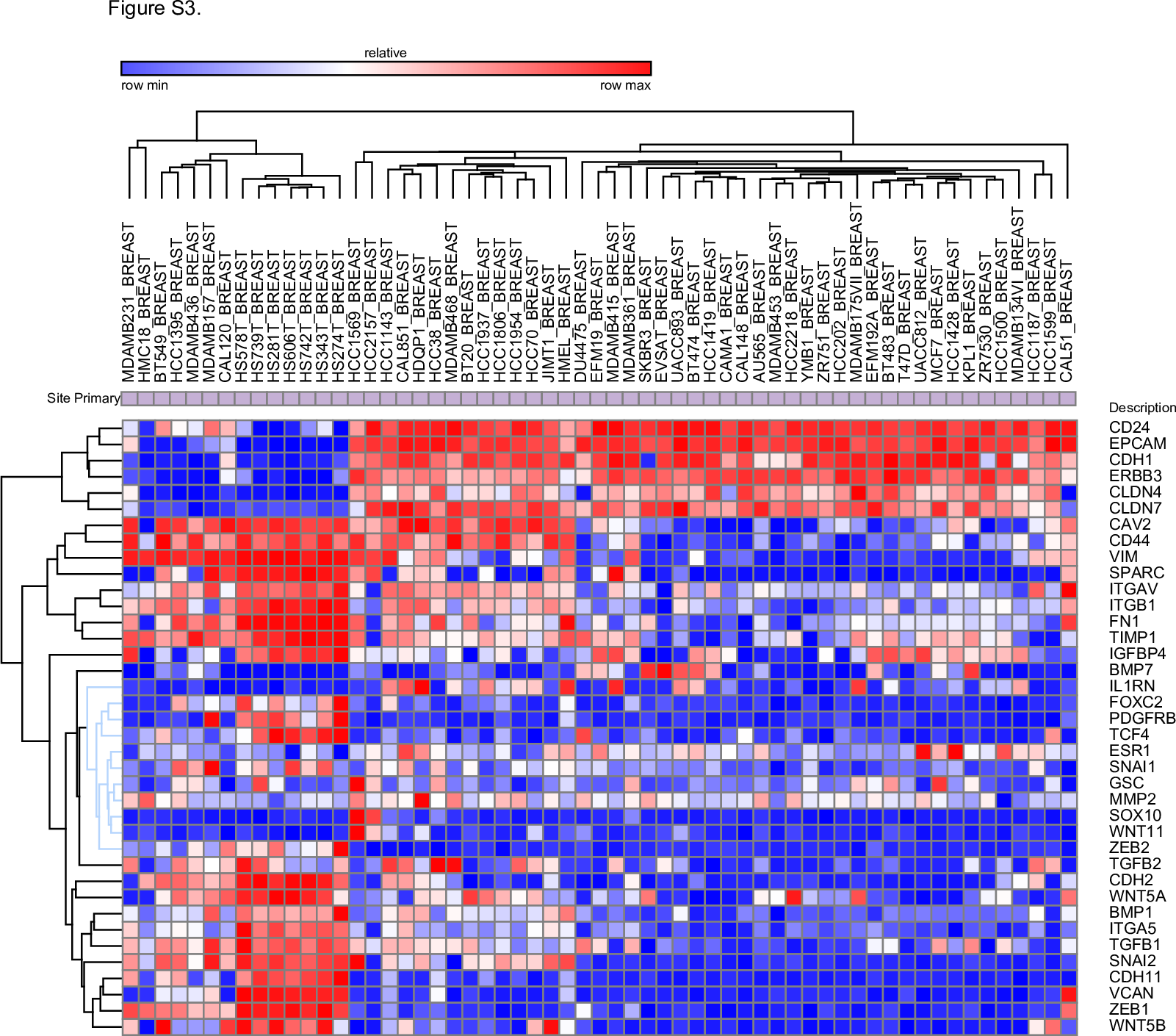

Supplement: S3 Fig — Panel of breast cancer cell line (https://sites.broadinstitute.org/ccle/) were hierarchically analyzed using Gene-E software to separate epithelial and mesenchymal cell lines based on EMT gene signature. Left side rows designate mesenchymal cells and right side is for epithelial cells. EpCAM and ZEB1 expression can be seen in two different populations. (TIF) [file pone.0285707.s003.tif]

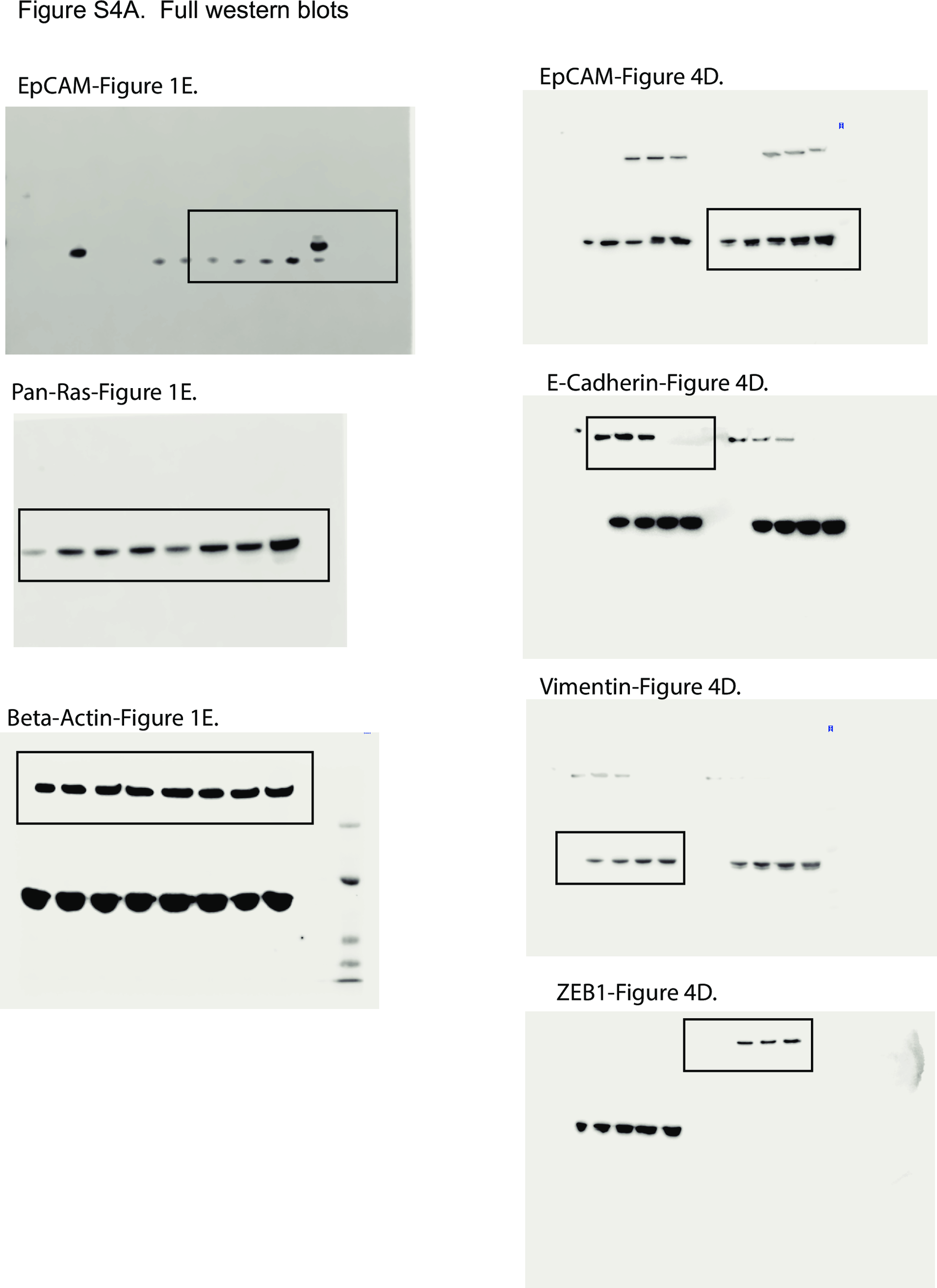

Supplement: S4 Fig — A. Full western blots. Full western blots supporting Figs 1E and 4D. B. Full western blots. Full western blots supporting Fig 4C. C. Full western blots. Full western blots supporting Fig 6B. D. Full western blots. Full western blots supporting Fig 6E. (ZIP) [file pone.0285707.s004.zip › Figure S4A.tif]

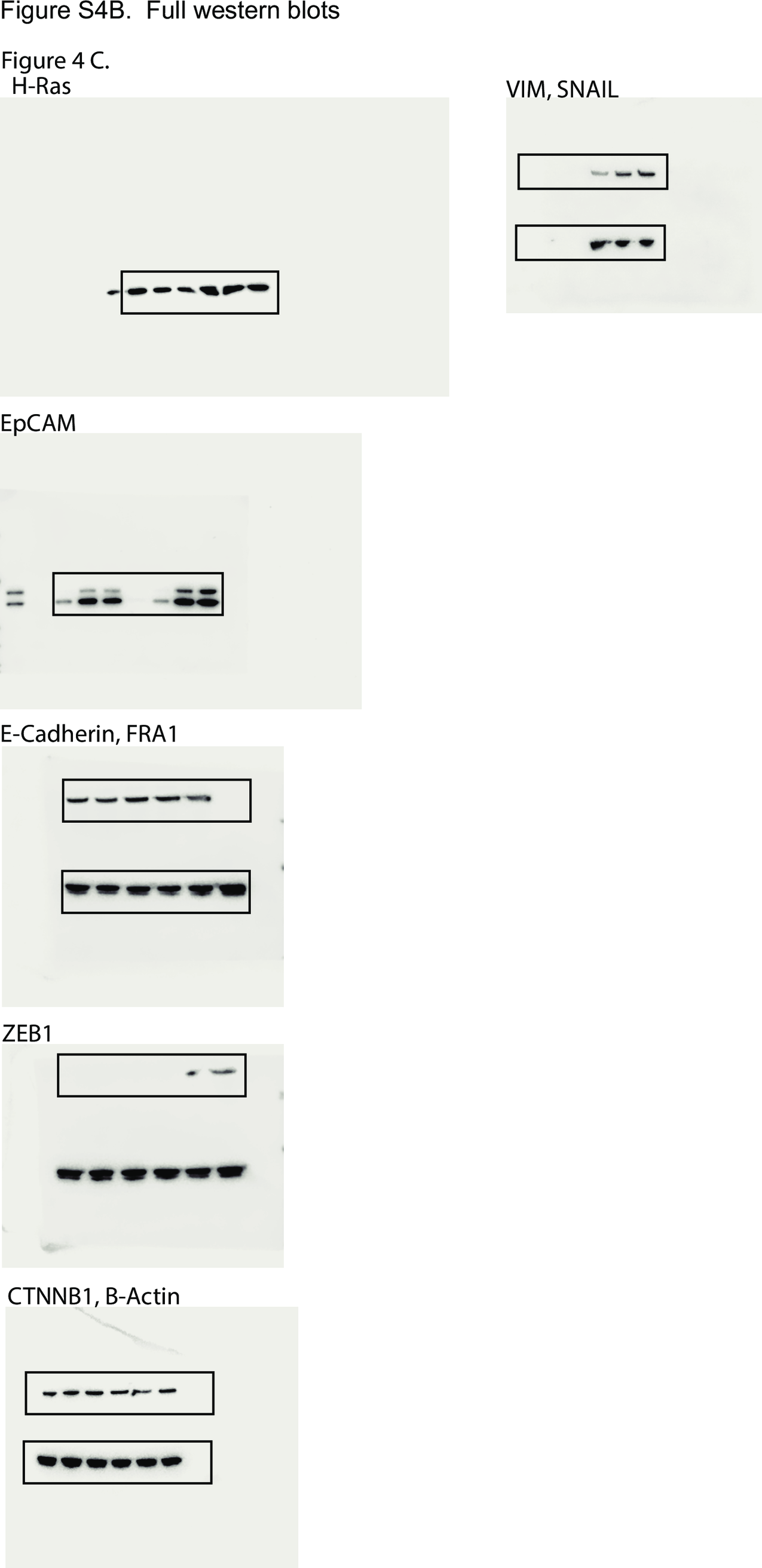

Supplement: S4 Fig — A. Full western blots. Full western blots supporting Figs 1E and 4D. B. Full western blots. Full western blots supporting Fig 4C. C. Full western blots. Full western blots supporting Fig 6B. D. Full western blots. Full western blots supporting Fig 6E. (ZIP) [file pone.0285707.s004.zip › Figure S4B.tif]

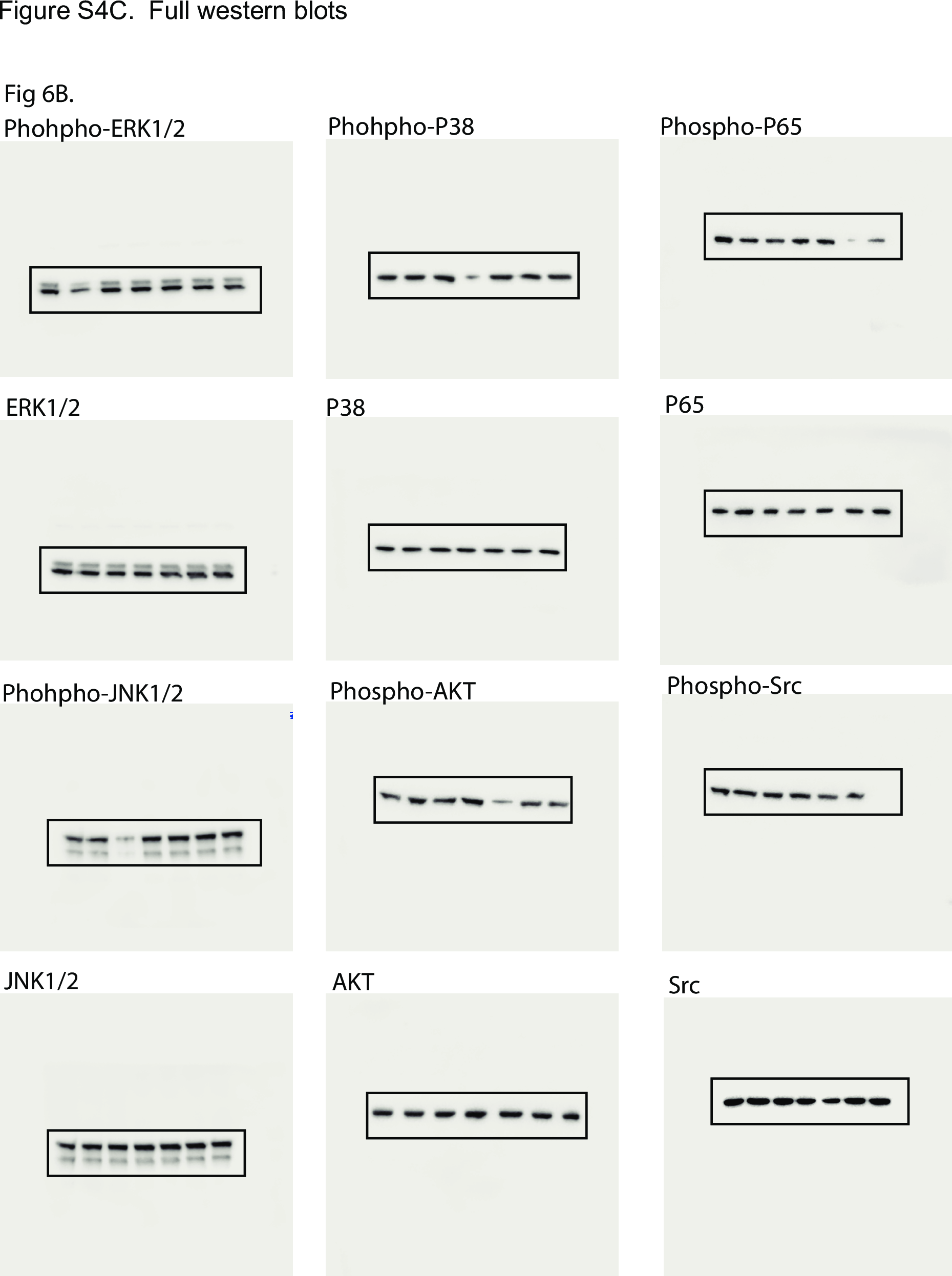

Supplement: S4 Fig — A. Full western blots. Full western blots supporting Figs 1E and 4D. B. Full western blots. Full western blots supporting Fig 4C. C. Full western blots. Full western blots supporting Fig 6B. D. Full western blots. Full western blots supporting Fig 6E. (ZIP) [file pone.0285707.s004.zip › Figure S4C.tif]

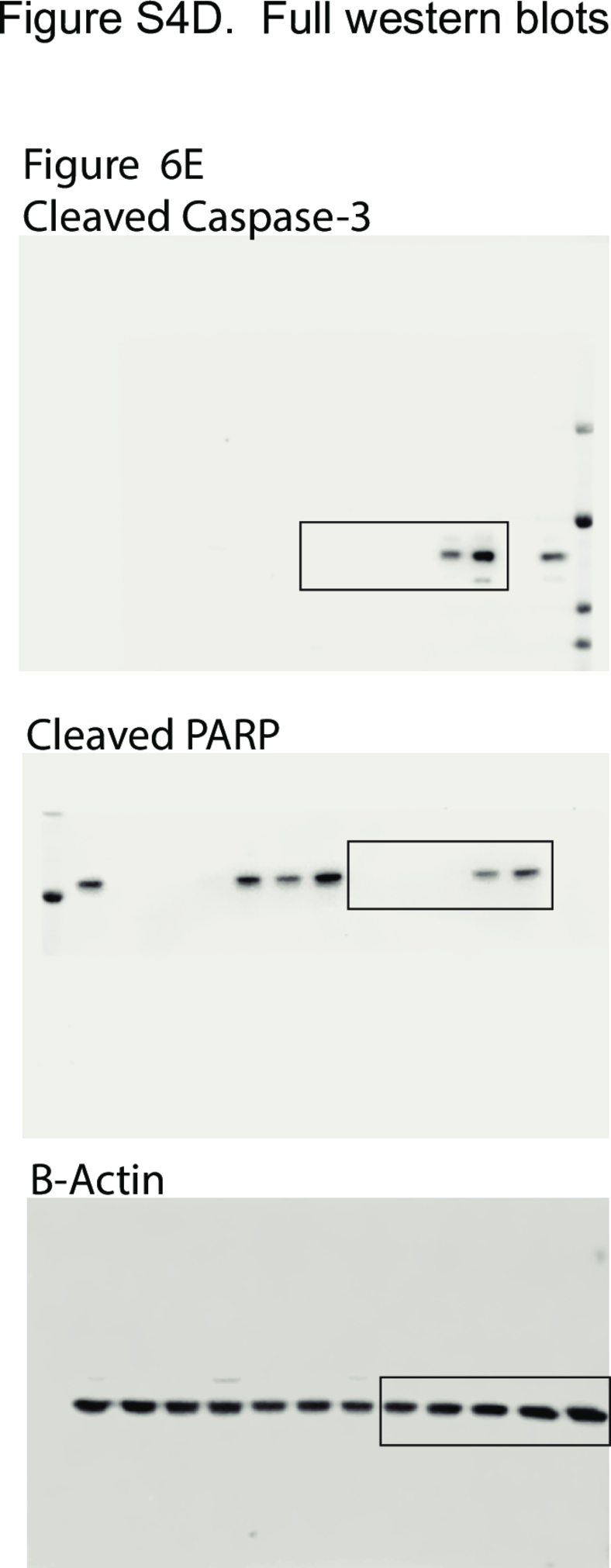

Supplement: S4 Fig — A. Full western blots. Full western blots supporting Figs 1E and 4D. B. Full western blots. Full western blots supporting Fig 4C. C. Full western blots. Full western blots supporting Fig 6B. D. Full western blots. Full western blots supporting Fig 6E. (ZIP) [file pone.0285707.s004.zip › Figure S4D.tif]
