# Supplementary material for: Cytosolic EpCAM cooperates with H-Ras to regulate epithelial to mesenchymal transition through ZEB1
Source: PLoS One. 2023 May 16;18(5):e0285707. doi: 10.1371/journal.pone.0285707 (PMC10187930; doi:10.1371/journal.pone.0285707)
Supplement: S1 Table — List of qRT-PCR primers. (DOCX) [file pone.0285707.s005.docx]

**Table S1. List of qRT-PCR primers**

| **Gene** | **Primer Forward** | **Primer Reverse** |
| --- | --- | --- |
| EpCAM | CTGCCAAATGTTTGGTGATG | ACGCGTTGTGATCTCCTTCT |
| CD24 | TGCTCCTACCCACGCAGATT | GGCCAACCCAGAGTTGGAA |
| E-Cadherin | CGGGAATGCAGTTGAGGATC | AGGATGGTGTAAGCGATGGC |
| Caludin-7 | CCTGGTATGGCCATCAGATT | GGACAGGAACAGGAGAGCAG |
| SPINT2 | CACGACTTCTGCCTGGTGT | GAGGCACTCCTCCTTGGTC |
| SLUG | GAGCATACAGCCCCATCACT | GGGTCTGAAAGCTTGGACTG |
| SNAIL | CGCGCTCTTTCCTCGTCAG | TCCCAGATGAGCATTGGCAG |
| TWIST1 | GTCCGCAGTCTTACGAGGAG | CCAGCTTGAGGGTCTGAATC |
| ZEB1 | GCACCTGAAGAGGACCAGAG | GTGTAACTGCACAGGGAGCA |
| ZEB2 | CGCTTGACATCACTGAAGGA | GATCTGTCCCTGGCTTGTGT |
| FRA1 | AGCTGCAGAAGCAGAAGGAG | GGAGTTAGGGAGGGTGTGGT |
| FRA2 | GGGTAGATATGCCTGGCTCA | GGTATGGGTTGGACATGGAG |
| FOCX2 | GATCACCTTGAACGGCATCT | TCTCCTTGGACACGTCCTTC |
| LOX | CAGAGGAGAGTGGCTGAAGG | CCAGGTAGCTGGGGTTTACA |
| VIM | CAGGCTCAGATTCAGGAACAG | CAGAGAGGTCAGCAAACTTGG |
| CD44 | CAACACAAATGGCTGGTACG | GTGTGGTTGAAATGGTGCTG |
| CD44v4 | CATCTACCCCAGCAACCCTA | CCCATGTGAGTGTCCATCTG |
| MMP1 | CTGGGAGCAAACACATCTGA | CTGCTTGACCCTCAGAGACC |
| MMP2 | TTGACGGTAAGGACGGACTC | CTCCCAAGGTCCATAGCTCA |
| MMP7 | CGGATGGTAGCAGTCTAGGG | TGGGGATCTCCATTTCCATA |
| MMP9 | CAGTCCACCCTTGTGCTCTT | CTATCCAGCTCACCGGTCTC |
| MMP11 | GGGGATGTCCACTTCGACTA | CAGTGGGTAGCGAAAGGTGT |
| AREG | CATTATGCTGCTGGATTGGA | AGCCAGGTATTTGTGGTTCG |
| TGFB1 | GGGACTATCCACCTGCAAGA | CCTCCTTGGCGTAGTAGTCG |
| IL-6 | CACACAGACAGCCACTCACC | GCCATCTTTGGAAGGTTCAG |
| IL-8 | GTGCAGTTTTGCCAAGGAGT | ACTTCTCCACAACCCTCTGC |
| CCL5 | CGCTGTCATCCTCATTGCTA | GAGCACTTGCCACTGGTGTA |
| 18srRNA | CCTGCGGCTTAATTTGACTC | GAACGCCACTTGTCCCTCTA |
| GAPDH | CGAGATCCCTCCAAAATCAA | GTCTTCTGGGTGGCAGTGAT |
