# Supplementary material for: Cytosolic EpCAM cooperates with H-Ras to regulate epithelial to mesenchymal transition through ZEB1
Source: PLoS One. 2023 May 16;18(5):e0285707. doi: 10.1371/journal.pone.0285707 (PMC10187930; doi:10.1371/journal.pone.0285707)
Supplement: Attachment — Submitted filename: PLOSONE PONE-D-22-34859 revision.docx [file pone.0285707.s006.docx]

In this manuscript, the authors investigated the role of cytosolic EpCAM in MCF-10A cell line modified to overexpress the H-Ras oncogene. The manuscript is well written and well structured, with several experiments aimed at defining the interconnected role of H-Ras overexpression and EpCAM in EMT. Figures and graphs are well defined.

There are only a few comments to further improve the work and make it even more complete.

**Point 1**: The role and possible collateral effects that targeted inhibition of EpCAM would have in epithelial tumors should be expanded.

**Point 2**: Did you analyze and define the type of cell death involved in the treatment with Paclitaxel and Quercetin? If yes, it would be interesting to add it in the results.

**Point 3**: In the materials and methods section please add more information about the inhibitors used to test the EpCAM-transformed cells.

**Point 4:** In the materials and methods section there are some subsections in which the number of cells seeded to perform the experiments has not been included. Please add them.

**Point 5**: Again, in the materials and methods section, expand the *statistical analysis* part.
